# Do pain, anxiety and depression influence quality of life for people with amyotrophic lateral sclerosis/motor neuron disease? A national study reconciling previous conflicting literature

**DOI:** 10.1007/s00415-019-09615-3

**Published:** 2019-11-07

**Authors:** Rhiannon Edge, Roger Mills, Alan Tennant, Peter J. Diggle, Carolyn A. Young, the TONiC study group, Ammar Al-Chalabi, Timothy L. Williams, David J. Dick, Kevin Talbot, Georgina Burke, Tahir Majeed, John Ealing, Christopher J. McDermott, Ashwin Pinto, Carolyn A. Young, Siddharthan Chandran, Jannette Walsh, Oliver Hanemann, Timothy Harrower

**Affiliations:** 1 Lancaster Medical School, Lancaster University, B38 Furness Building, Bailrigg, Lancaster, UK LA1 4YW; 2 Walton Centre NHS Trust, Lower Lane, Liverpool, L9 7LJ UK; 3 University of Liverpool, Liverpool, L69 3BX UK; 4 Emeritus Professor, Leeds Institute of Rheumatic and Musculoskeletal Medicine, University of Leeds, Leeds, UK

**Keywords:** Amyotrophic lateral sclerosis (ALS), Motor neuron disease (MND), Quality of life, TONiC study, Anxiety, Depression, Pain

## Abstract

**Introduction:**

The importance of elucidating the relationships between pain, mood and quality of life (QoL) amongst people with amyotrophic lateral sclerosis/motor neuron disease is evident to clinicians, yet the literature is limited and inconsistent. We explored the relationships between pain, depression, anxiety and QoL to reconcile the previous contrasting findings and inform future research and clinical practice.

**Methods:**

Patient-reported outcomes were obtained as part of the Trajectories of Outcomes in Neurological Conditions study. Mood and QoL scales underwent Rasch analysis. Correlation coefficients examined the strength of association between variables of interest. A bivariate regression model was developed to examine the effects of pain, depression and anxiety on joint psychological and physical QoL domains.

**Results:**

Of 636 people with ALS, 69% reported pain, of these most had mild pain. Seven percent (7%) of participants exceeded published cutoffs for probable depression and 14% had probable anxiety. Pain, depression and anxiety all influence quality of life; depression has a significant effect on both physical and psychological domains of QoL, whereas pain affects physical QoL and anxiety psychological QoL.

**Conclusions:**

These results show the importance of expressing quality of life in a conceptually appropriate way, as failing to take account of the multidimensional nature of QoL can result in important nuances being overlooked. Clinicians must be aware that pain, depression and anxiety all worsen QoL across their ranges, and not just when pain is severe or when anxiety or depression reach case level.

**Electronic supplementary material:**

The online version of this article (10.1007/s00415-019-09615-3) contains supplementary material, which is available to authorized users.

## Introduction

Amyotrophic lateral sclerosis (ALS), also known as motor neuron disease (MND), is a progressive, neurodegenerative disease leading to paresis, dysarthria, dysphagia, respiratory failure and death. No curative treatment is currently available, so the paramount clinical consideration is symptom relief to improve quality of life (QoL) [1]. Since QoL for people with ALS/MND is shaped not only by the physical manifestations of the disease, but also by the psychosocial effects of living with a terminal, disabling illness, these symptoms range from physical aspects such as pain to psychological aspects such as depression and anxiety [2].

A recent review highlighted pain as a symptom which is poorly understood amongst ALS/MND populations [3]. The hypothesis that pain would reduce QoL was supported by one study of 40 patients with ALS/MND [4] but refuted by another study analysing 36 patients, which found that the effect of pain intensity upon QoL was no longer significant once depression scores were added as a possible confounding covariate [5].

Psychological distress, including depression and anxiety, might be predicted to worsen QoL among those with ALS/MND but again the literature is conflicting. While there are a number of studies reporting that depression does adversely affect QoL [5–12], there are many finding no correlation [2, 13–15] or variable findings [16–18]. Similarly, the literature on anxiety ranges from those linking anxiety with a worse QoL [19–21] to several reporting no correlation [2, 14, 15]. These contrasting findings have not yet been explained; speculation that small sample sizes may have led to unrepresentative cohorts is refuted by variation regarding the effect of depression on QoL among studies of at least 100 patients, where again there are conflicting results finding negative [7, 10, 11] or no correlation [13]. Consideration that choice of outcome measure for depression or anxiety may be responsible for these contrasting results is contradicted by different conclusions even when the same measure is used. Studies on the relation between anxiety and depression with QoL in ALS/MND using the Hospital Anxiety and Depression scale (HADS) have shown an association between anxiety and QoL [21], or depression and QoL [10], or conversely no relation for either anxiety or depression [2, 14].

The ongoing Trajectories of Outcomes in Neurological Conditions (TONiC) study in people with ALS/MND examines the relationships between QoL and a number of potential factors which could be influenced by clinical care. TONiC-ALS involves a number of phases and began with qualitative work with people with ALS/MND, through 1:1 interviews and focus groups, to identify which factors people living with the condition believed influential on their QoL. To ascertain which factors are believed amenable to clinical care and to increase the likelihood that findings would translate to practice, we followed the patient-centred, qualitative work with a nationally promoted, online forum for health and social care professionals to ascertain the face validity of the patient findings among professionals [22]. These processes showed that despite the variation in the literature findings, patients and professionals alike believe that pain, anxiety and depression all influence QoL for people with ALS/MND. People with ALS/MND described their QoL as involving physical, psychological and other aspects, in keeping with the World Health Organization’s recognition of the multidimensional nature of QoL [23].

The aim of this paper is to examine the prevalence of pain, anxiety, and depression in a large sample of people with ALS/MND, and examine their inter-relationships and effect on QoL, utilising a measure that recognises the multi-faceted nature of QoL. We also sought to reconcile the previous contrasting findings to inform future research.

## Methods

### Data collection

Participants with ALS/MND, diagnosed according to El Escorial World Federation of Neurology criteria for the diagnosis of ALS [24], were recruited into the ongoing TONiC study from specialist clinics across the United Kingdom using a convenience sampling strategy. Cases with a family history of ALS/MND were eligible as were patients with only lower motor neuron (LMN) signs in two or more regions, or with progressive primary lateral sclerosis without spinal LMN signs, provided a consultant neurologist specialising in ALS/MND had confirmed the diagnosis. Eligibility criteria were wide and inclusive, permitting participants to join irrespective of level of disability, duration of disease or subtype of MND (amyotrophic lateral sclerosis, progressive bulbar palsy, progressive muscular atrophy, primary lateral sclerosis). Participants were excluded if they were unable to give informed consent or unable to complete self-report questionnaires even with help from a scribe. Participants were not eligible if they had cognitive impairment precluding them being able to select responses on patient-reported outcomes (PROs) or if they had significant concomitant conditions which would have influenced their answers to the PROs. Inclusion and exclusion criteria were assessed by a specialist healthcare worker prior to enrollment in the study. Not all approached patients agreed to participate, typically because of perceived lack of time or because they preferred not to think about MND and its effects. Following informed consent, participants completed a questionnaire pack containing demographic and disease-specific data such as duration from diagnosis (all UK MND clinics follow rapid diagnosis pathways), as well as a range of patient-reported measures [25].

The measures included:

1. World Health Organization Quality of Life questionnaire (WHOQOL-BREF) [26]—the scale measures generic quality of life across four domains: physical health (seven items, with operational score range of 0–28), psychological health (six items, with operational score range 0–24), environment and social relationships [23]. The physical health domain was designed to incorporate pain, amongst other factors, whereas it was intended that the psychological health domain would incorporate positive and negative feelings, amongst other factors.
2. Hospital Anxiety and Depression Scale for MND (mHADS) [27]—a version of the HADS has been previously validated by Rasch analysis for use in an MND population, with removal of two items from the original 14-item scale. The modified summed raw-score cut-points of between five and seven indicate a possible case, and above or equal to eight indicate a probable case of depression. Similarly, scores between seven and eight indicate a possible case, and above or equal to nine indicate a probable case for the anxiety scale.
3. Pain—this was measured by a numeric rating scale (NRS), anchored as 0—no pain and 10—severe pain, to describe overall pain level. Pain was treated as a continuous variable in the analysis.

### Rasch analysis

Interval level estimates for each person were calculated for the anxiety and depression scales of the mHADS and the WHOQOL-BREF physical and psychological domains, by Rasch analysis (see supplementary) [28, 29]. The explained common variance (ECV) is reported where a value of 1 indicates that all non-error variance is contained within the latent estimate. An ECV value > 0.9 is considered sufficient to indicate that the first common factor is essentially unidimensional [30]. Full details of this process are given elsewhere [31–33]. The resulting interval level transformations were translated to the original operational range of each scale.

### Other statistical analyses

Summary statistics were produced as part of the initial data investigation. Pearson’s (or where appropriate Kendall’s) correlation coefficients were calculated to examine the strength of association between variables of interest. A type 2 MANOVA was used to test the null hypothesis that there is no effect of pain, anxiety, and depression on the joint distribution of physical and psychological QoL. Following this investigation, a statistical modelling analysis was performed to assess the joint effects of pain, depression and anxiety on the psychological and physical QoL domains.

Our model is an extension of the standard linear regression model by accommodating two dependent variables, which are assumed to follow a bivariate normal distribution. The dependent variables are physical and psychological domains of QoL and the independent variables are pain, depression and anxiety. Bivariate models of this kind are able to take account of correlations amongst the dependent variables and so can be more powerful than separate multiple linear regressions for each variable [34]. A final model was selected following a process of forwards selection using the Pillai’s trace statistic [35], and interaction effects between significant variables were tested for inclusion.

The results are shown in a coefficient plot, which illustrates the effects of each independent variable on the dependent variables by showing the parameter estimates and confidence ellipses for each coefficient in the model. The variables are standardised so that their corresponding regression parameter estimates can be compared directly. A joint, multivariate test of H_0_: *β* = zero is rejected when the confidence ellipse does not cover the origin (labelled as H_0_ on the plot). The size of each ellipse corresponds to the confidence in the parameter estimate whereby in 95% analyses, the ellipse will contain the true (but unknown) value of the parameter estimate.

## Results

### Participants

636 participants completed the questionnaire pack at time of analysis (Table 1).

**Table 1:** Descriptive statistics for the cohort

*N*	636
Age/years [mean (SD)]	65.12 (10.74)
Duration from diagnosis/months (median [IQR])	10.0 [4.0, 26.0]
Onset type (%)	
Bulbar	173 (27.2)
Limb	432 (67.9)
Respiratory	12 (1.9)
Unknown	19 (3.0)
Sex = M (%)	390 (61.3)

### Rasch analysis

Data from the physical and psychological domains of the WHOQOL-BREF and the anxiety and depression scales all fitted the Rasch model well, with non-significant total Chi square values. Reliability indicators were > 0.70, the conventional threshold for acceptability for group use. All ECV values were ≥ 0.9. Detailed summary fit statistics can be found in the supplementary material (Table S1).

### Pain

**Fig. 1:**
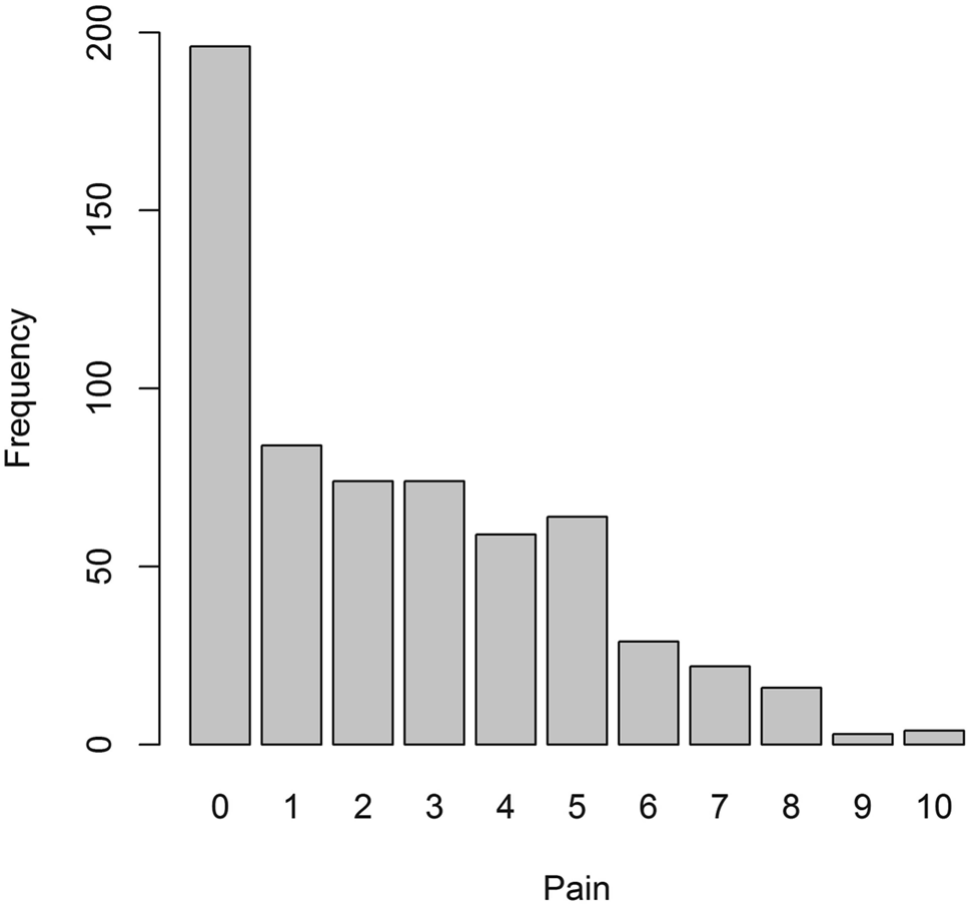
A bar chart showing the responses to the pain numerical rating scale

The pain NRS was completed by 625/636 (98.3%) individuals, of whom 429 (68.6%) reported pain by giving a score above the zero anchor of no pain (Fig. 1). Median score was 2 (IQR 2–4).

### Mood

Mean depression measurement was 5.50 (SD 3.45) and mean anxiety measurement was 5.49 (SD 3.17). The proportions of participants with scores above the cut-points that indicate a possible or probable clinical case were: 29.1% for depression and 27.4% for anxiety. The proportions categorised as probable cases were much lower (Table 2).

**Table 2:** The modified cutoffs from the mHADS for depression and anxiety

	Unlikely cases	Possible cases	Probable cases	Total
Anxiety (%)	458 (72.6%)	85 (13.5%)	88 (13.9%)	631
Depression (%)	446 (70.9%)	137 (21.8%)	46 (7.3%)	629

### Correlations between pain, mood and quality of life

597 individuals provided complete information about their pain, mood (mHADS anxiety and depression) and QoL. There were some strong correlations between these variables, for example, physical QoL was associated with psychological QoL (Table 3). Pain was most strongly correlated with physical QoL, whereas depression and anxiety were most strongly correlated with psychological QoL.

**Table 3:** Correlation matrix of the main variables of interest

	Pain	Depression	Anxiety	Physical QoL
Depression	0.212			
Anxiety	0.173	0.581		
Physical QoL	− 0.373	− 0.528	− 0.364	
Psychological QoL	− 0.191	− 0.726	− 0.615	0.601

Kendall’s tau correlation coefficients are provided for the variables that are associated with pain, and Pearson’s correlation coefficients are given for the remaining associations

### Statistical model of physical and psychological quality of life domains

Acknowledging the strong correlation between physical and psychological QoL, we explored the influence of pain, depression and anxiety on their joint distribution. A type 2 MANOVA was used to test the null hypothesis that there is no effect of pain, anxiety, and depression on the joint distribution of physical and psychological QoL. Interactions between the main effects were tested for, and an interaction effect between pain and depression was found to be significant. The null hypothesis was rejected for each explanatory variable (Table 4). Depression explains more of the variance in the response variables than the other explanatory variables (Pillai test statistic = 0.33).

**Table 4:** Results of the MANOVA of pain, anxiety and depression on physical and psychological QoL

Variable	Degrees of freedom	Pillai’s trace test statistic	Approximate *F* statistic	*df*_1_^a^	*df*_2_^a^	*p* value
Pain	1	0.184	69.143	2	612	< 0.0001
Depression	1	0.328	149.165	2	612	< 0.0001
Anxiety	1	0.122	42.623	2	612	< 0.0001
Pain × depression	1	0.024	7.497	2	612	< 0.001

^a^*df*_1_ and *df*_2_ are the degrees of freedom used in determining the *F* value i.e. *F*(*df*_1_, *df*_2_)

### Model selection

A final model including pain, depression, anxiety and an interaction between pain and depression was identified. Each parameter is significant in the bivariate model for physical and psychological QoL (Table 5). Increased pain, depression, and anxiety are associated with a reduction in QoL. The positive coefficient for the interaction term between pain and depression suggests that there is a less than additive effect on QoL for those with both variables. Depression is the most influential factor for both QoL domains.

**Table 5:** Standardised model parameters for the bivariate regression model of physical and psychological QoL, *N* = 618

	Standardised parameter estimate	Std. error	*t* value	Pr(> |*t*|)
Physical QoL				
Intercept	14.467	0.121	119.868	< 2e−16
Pain	− 1.434	0.123	− 11.679	< 2e−16
Depression	− 1.493	0.146	− 10.241	< 2e−16
Anxiety	− 0.210	0.144	− 1.457	0.146
Pain × depression	0.442	0.116	3.821	< 0.001
Psychological QoL				
Intercept	13.596	0.088	154.235	< 2e−16
Pain	− 0.112	0.090	− 1.246	0.213
Depression	− 1.783	0.106	− 16.753	< 2e−16
Anxiety	− 0.946	0.105	− 8.986	< 2e−16
Pain × depression	0.074	0.085	0.870	0.385

### Coefficient plot

**Fig. 2:**
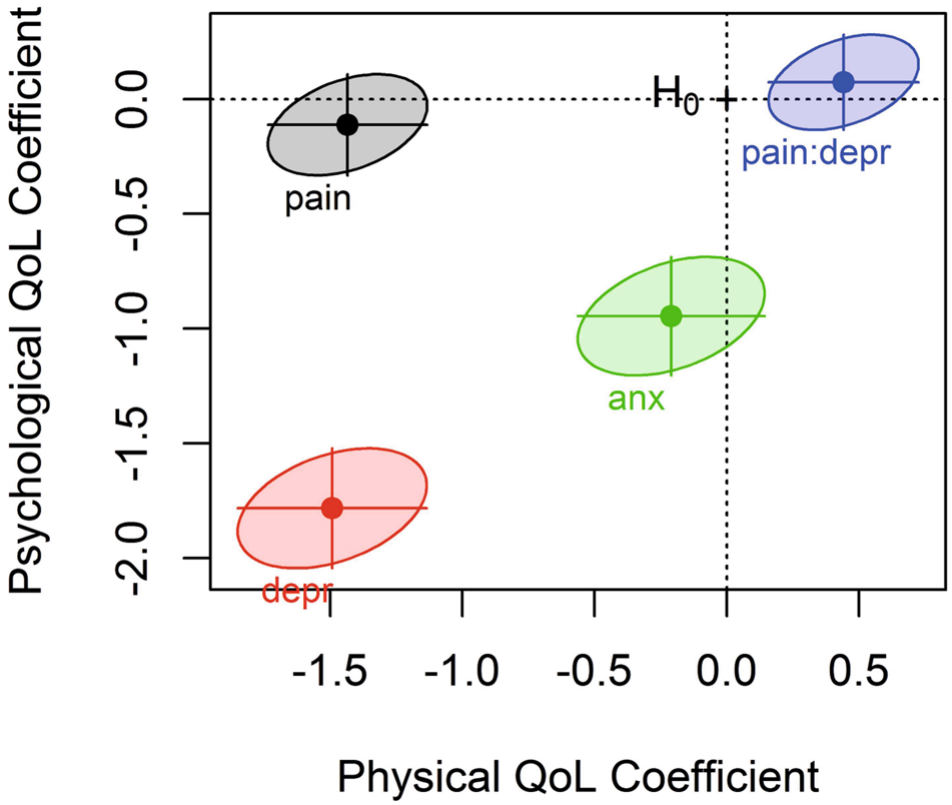
Coefficient plot for the parameters of the bivariate model

The coefficient plot provides a graphical interpretation of the results from the bivariate regression analysis (Fig. 2). It shows that all three factors of pain, depression and anxiety influence the bivariate model of physical and psychological QoL, as the confidence ellipse for each one does not cross the origin (H_0_). Note that the confidence ellipses for pain, and for the interaction term between pain and depression, include the zero value for psychological QoL, and that the confidence ellipse of anxiety includes the zero value for physical QoL.

## Discussion

Pain is common in a large, national sample of people living with ALS/MND, reported by 429/625 (68.6%). Across a numerical rating scale anchored between no pain (0) and severe pain (10), about half reported values of 1–4, and less than 5% reported values of 8–10. Like pain, mood is a continuum, which can be divided into probable, possible or unlikely cases according to the number and severity of depression or anxiety symptoms endorsed in the HADS [27]. Depression and anxiety were common; 7% had probable depression, and 14% had probable anxiety, according to their mHADS. There were more participants who had possible depression (21.8%) and a smaller number with possible anxiety (13.5%). Many participants were unlikely to have anxiety (72.6%) or depression (70.9%). Using a bivariate statistical model, we were able to better elucidate the joint effects of pain and mood on the different domains of QoL. We described QoL in a conceptually appropriate way, using the joint distribution of psychological and physical QoL. The bivariate model shows that pain, depression, and anxiety affect physical and psychological QoL.

Previous smaller studies have found comparable prevalence of pain in ALS: 53.8% in a multicentre study (*n* = 80) [36], 56.9% in a regional study (*n* = 160) [37], and 78–85% in two small clinic-based studies (*n* = 46 or *n* = 42) [38, 39]. Those studies including a control group showed that pain in ALS/MND exceeded the population-based control rate [37–39]. The distribution of pain severity in our study is similar to an ALS patient online survey of 2664 respondents which found 39% report ‘none’, 34% report ‘mild’, and 6% report ‘severe’ [40]. A 2013 literature review of published research stated that the prevalence of pain in older people living in the community ranged from 20 to 46% [41]. Boer et al. used the NRS to assess pain amongst the general population (*n* = 376; 89 (65.0%) men, mean age 53.2 years). Of the respondents, 36.4% (*n* = 137) reported pain complaints, with a mean NRS pain score of 4.65 (standard deviation (SD) = 2.05, range 1–9) [42]. Our large study showed that the prevalence of pain in those with ALS/MND is higher than the figures given above for the general population, although the pain intensity for those with pain is similarly mild.

This study highlights the importance of quantifying QoL in an appropriate way. Considering both physical and psychological aspects of QoL in ALS/MND jointly facilitated a greater understanding of how pain, depression and anxiety affect QoL. Pain, depression and anxiety influence different domains of QoL, as was proposed a priori when the WHOQOL-BREF was developed [23]. The model shows that pain influences physical QoL more than psychological QoL, whereas depression has a large influence on both physical and psychological QoL. Anxiety is highly correlated with psychological QoL and mildly correlated with physical QoL. Earlier work suggested that pain was significantly correlated to overall QoL, physical wellbeing and existential wellbeing, but not psychological wellbeing [4]. A literature search on the relationships between pain, mood and QoL in ALS shows just one other paper reporting a linear regression model investigating pain, depression and QoL in a sample of 36 ALS patients [5]. They found a weak association between increasing pain and lower QoL, which was no longer significant once depression was accounted for. The QoL measure used was the Spitzer QoL index [43], which is reported as a single number between 0 and 10. Using a multidimensional description of QoL in our model allows a fuller depiction of QoL compared to a single-item measure [18].

Many of the earlier studies which also found negative correlations between QoL and depression [6–8, 10], or anxiety [19–21] used multidimensional QoL measures. Those studies not finding correlations between QoL with depression or anxiety used single-item measures of QoL [13], or subject-specific measures, where respondents nominate and indicate the relative importance of QoL domains [2, 14, 16]. Using regression analyses, Sandstedt et al. found that depression and anxiety, as assessed by the HADS, were associated with the psychosocial score rather than physical score in the Sickness Impact Profile measure for QoL [18].

It has been suggested that anxiety often co-exists with depression in ALS [44], which may lead to over-reporting of the contribution of anxiety to QoL in analyses where depression has not been accounted for. This incongruity stresses the importance of interpreting statistical analyses with care. For example, using the TONiC dataset, we performed a simple multiple regression of the effects of anxiety, depression and pain on overall QoL (see supplementary); anxiety was not significant in the multiple regression model for overall QoL once pain and depression had been accounted for. In view of our large sample size, this result is likely to be due to the high correlations amongst pain, depression and anxiety. In the bivariate model for physical and psychological QoL, anxiety remained a significant contributor even after pain and depression were accounted for. These findings are in keeping with a review concluding that higher levels of anxiety and depression were associated with poorer QoL in ALS/MND [1]. Our final model included a positive interaction term between pain and depression; for those with both depression and pain, the effect on QoL is slightly less than the additive effect that might otherwise have been inferred from a model that does not account for co-existent symptoms.

The strengths of this work include the large sample size (*N* = 625) collected nationally, that pain and mood information were collected as part of a wider assessment so that the data reflect general patient experience, and the use of a QoL measure which reflected the viewpoint of people with ALS/MND that their QoL was influenced by physical, psychological and other components. The limitations of this work are its cross-sectional design so causality cannot be determined. A longitudinal extension providing repeated measurements over time is under way to allow these aspects to be investigated. Patients with cognitive impairment were ineligible to participate in this study and the findings of this study will not necessarily be relevant for ALS/MND patients with cognitive impairment. Minor cognitive impairment amongst participants was not assessed, and further work could aim to explore any effect of cognitive impairment on the relationship between pain, depression and anxiety on QoL.

This study highlights the adverse effects of pain, depression, and anxiety on QoL for those living with ALS/MND. Clinicians should routinely enquire about pain as they do for other symptoms such as orthopnea or emotional lability. Pain offers a potentially important treatment target since this symptom influences quality of life, has many treatments, and is common, with almost 70% of people with ALS/MND experiencing some level of pain. The Practice Parameters of the American Academy of Neurology [45] and the European Guidelines on the Clinical Management of ALS [46] discuss the treatment of pain for patients with ALS/MND and the topic has been extensively reviewed [3].

There is consensus among experts that patients with ALS/MND who are diagnosed with either depression or anxiety may require treatment but both the American and European guidelines note the absence of systematic trials [45, 46]. Guidelines comment that the choice of antidepressant may be influenced by additional symptoms (e.g. sialorrhoea, insomnia, apathy, appetite loss), which are differently affected by the various antidepressants, or utilise the anxiolytic effects of some antidepressants to treat both depression and co-existing anxiety [46].

While clinicians may prefer to defer pharmacotherapy for those patients with symptoms of depression or anxiety whose screening scores are below the cut-points for diagnosis as probable cases, such patients should be monitored for later deterioration. It is important to note that the adverse effects of depression and anxiety each played out across the spectrum of severity; so restricting treatment or additional support only to those patients who are categorised as probable cases disregards the negative impact of milder depression or anxiety. Non-pharmacotherapeutic treatments such as acceptance and commitment therapy [47] or mindfulness [48] may be useful options. Depression emerges as an important factor meriting particular clinical vigilance and willingness to intervene, because it has a strong association with both physical and psychological QoL.

In conclusion, quality of life for people living with ALS/MND is a dynamic, multidimensional construct and this should be accounted for during research and care. Systematic and repeated assessments of pain, depression and anxiety emerge as worthwhile additions to comprehensive care, because these are common and treatable symptoms which impact upon the QoL of those living with ALS/MND.

## Electronic supplementary material

Below is the link to the electronic supplementary material.

Supplementary material 1 (DOCX 172 kb)
