# Correction to: Do pain, anxiety and depression influence quality of life for people with amyotrophic lateral sclerosis/motor neuron disease? A national study reconciling previous conflicting literature

**DOI:** 10.1007/s00415-020-09700-y

**Published:** 2020-01-28

**Authors:** Rhiannon Edge, Roger Mills, Alan Tennant, Peter J. Diggle, Carolyn A. Young, the TONiC study group, Ammar Al-Chalabi, Timothy L. Williams, David J. Dick, Kevin Talbot, Georgina Burke, Tahir Majeed, John Ealing, Christopher J. McDermott, Ashwin Pinto, Carolyn A. Young, Siddharthan Chandran, Jannette Walsh, C. Oliver Hanemann, Timothy Harrower

**Affiliations:** 1 Lancaster Medical School, Lancaster University, B38 Furness Building, Bailrigg, Lancaster, UK LA1 4YW; 2 Walton Centre NHS Trust, Lower Lane, Liverpool, L9 7LJ UK; 3 University of Liverpool, Liverpool, L69 3BX UK; 4 Emeritus Professor, Leeds Institute of Rheumatic and Musculoskeletal Medicine, University of Leeds, Leeds, UK

## Correction to: Journal of Neurology 10.1007/s00415-019-09615-3

The original version of this article unfortunately contained a mistake. C Oliver Hanemann's name was incorrect in the acknowledgements section of this paper.

The correct name should be C. Oliver Hanemann. The correct acknowledgements section should read as:

### Acknowledgements

The authors would like to thank the participants of the Trajectories of Outcome in Neurological Conditions (TONiC) Study for contributing their data for research.

**TONiC Study Group Membership**

Ammar Al-Chalabi^1^, Timothy L Williams^2^, David J Dick^3^, Kevin Talbot^4^, Georgina Burke^5^, Tahir Majeed^6^, John Ealing^7^, Christopher J. McDermott^8^, Ashwin Pinto^9^, Carolyn A. Young^10^, Siddharthan Chandran^11^, Jannette Walsh^12^, C. Oliver Hanemann^13^, Timothy Harrower^14^

*Affiliations*

1. King’s College London, London, UK

2. Royal Victoria Infirmary, Newcastle upon Tyne, UK

3. Norfolk and Norwich University Hospital, UK

4. University of Oxford, Oxford, UK

5. Queen Alexandra Hospital, Portsmouth, UK

6. Lancashire Teaching Hospitals, Preston, UK

7. Salford Royal Foundation Trust, Manchester, UK

8. University of Sheffield, Sheffield, UK

9. Wessex Neurological Centre, Southampton, UK

10. Walton Centre NHS Foundation Trust, Liverpool, UK

11. Royal Infirmary of Edinburgh, Edinburgh, UK

12. Staffordshire and Stoke on Trent Partnership NHS Trust, Newcastle-under-Lyme, UK

13. University of Plymouth, Drake Circus, Plymouth, UK

14. Northern Devon Healthcare NHS Trust, Devon, UK

*Email addresses*

1. ammar.al-chalabi@kcl.ac.uk

2. Tim.Williams@nuth.nhs.uk

3. david.dick@nnuh.nhs.uk

4. kevin.talbot@ndcn.ox.ac.uk

5. georgina.burke@porthosp.nhs.uk

6. Tahir.Majeed@lthtr.nhs.uk

7. john.ealing@srft.nhs.uk

8. c.j.mcdermott@sheffield.ac.uk

9. Ashwin.Pinto@uhs.nhs.uk

10. carolyn.young@thewaltoncentre.nhs.uk

11. siddharthan.chandran@ed.ac.uk

12. Jannette.Walsh@ssotp.nhs.uk

13. oliver.hanemann@plymouth.ac.uk

14. timothy.harrower@nhs.net

The members of the TONiC Study group contributed to the development of a larger programme of work of which this study is a part. The members have co-ordinated data collection as part of the development of the TONiC study. The TONiC study group’s contribution should be acknowledged as a corporate author.

