# Supplementary material for: Do pain, anxiety and depression influence quality of life for people with amyotrophic lateral sclerosis/motor neuron disease? A national study reconciling previous conflicting literature
Source: J Neurol. 2019 Nov 7;267(3):607–15. doi: 10.1007/s00415-019-09615-3 (PMC7035222; doi:10.1007/s00415-019-09615-3)
Supplement: Supplementary file 1 — Supplementary material 1 (DOCX 172 kb) [file 415_2019_9615_MOESM1_ESM.docx]

**Supplementary**

**Pain, mood and quality of life in Amyotrophic Lateral Sclerosis**

**1.1. Rasch analysis**

*Methods*

Rasch analysis is a probabilistic modeling technique that is now used as standard for assessing data derived from multi-item scales such as patient reported outcomes.^1^ Where data are shown to accord with model assumptions, and the validity of the scale can be supported, it is possible to transform ordinal, summed raw-score scale data into interval measurement.^2^ The Rasch Unidimensional Measurement Model 2030 (RUMM2030) software was used for each analysis, utilizing the partial credit parametrization of the model.^3,4^

Data from a scale are fit to the model, with various fit statistics available to test if the data satisfy the model requirements. A summary Chi-Square interaction fit statistic should be non-significant if there is no deviation between observed and expected scores across the trait being measured. A Person Separation reliability is reported, which is equivalent to alpha where there is a normal distribution, but otherwise deviates with skewed data.

Group invariance is tested through Differential Item Functioning (DIF) where estimates should be the same across groups, conditioned on the trait under consideration.^5^

Where a breach of the local independence assumption among items was found, a two-testlet approach was adopted to obviate local dependency and achieve fit of the data.^6^ Under this approach, a bi-factor equivalent solution is obtained with a reported Expected Common Variance (the ‘A’ values in RUMM 2030), where values ≥0∙9, are considered satisfactory and consistent with a unidimensional scale.^7,8^ Furthermore, a conditional Chi-Square test of fit becomes available which, again, should be non-significant.

Rasch analysis cannot be performed on single indicator scales such as the NRS

*Results*

Fit of scale data to the Rasch model was achieved by using a two-testlet approach.^9^ Testlets comprised the first three and last three items of the anxiety scale, first four and last two items of the depression scale, and alternative items for the WHOQOL domains. Fit statistics are provided in Table S1.

The WHOQOL-BREF has four sub-domains: physical, psychological, social relationships and environment and were not envisaged to be summated into a total score. Previous analysis of the domain structure of the WHOQOL-BREF in another patient group (post-polio syndrome) suggested that a higher order construct of QoL derived from combining the four domains could be valid.^10^

A bifactor solution (two testlets based on alternative items) to the four sub-domains in combination, was shown to be valid for an MND population, with satisfactory fit statistics (Table S1). Thus a holistic measurement of QoL could be used for subsequent analyses.

| PROM name | number of items | item residual mean | item residual SD | Person residual mean | Person residual SD | ChiSq p value | ChiSq value | DoF | PSI testlets | A | Cronbach alpha | conditional ChiSq p value | conditional ChiSq value | conditional ChiSq DoF | T test binomial lower CI (Agresti-Coull) |
| --- | --- | --- | --- | --- | --- | --- | --- | --- | --- | --- | --- | --- | --- | --- | --- |
| HADS-MND Anxiety | 6 | 0.219 | 1.360 | -0.505 | 0.856 | 0.411 | 18.695 | 18 | 0.768 | 0.994 | 0.838 | 0.175 | 13.958 | 10 | 0.018 |
| HADS-MND Depression | 6 | -0.248 | 3.067 | -0.412 | 0.755 | 0.109 | 20.705 | 14 | 0.679 | 1.008 | 0.740 | 0.142 | 10.918 | 7 | 0.018 |
| WHOQOL physical | 7 | 0.125 | 2.066 | -0.584 | 0.920 | 0.998 | 5.299 | 18 | 0.806 | 1.010 | 0.794 | 0.373 | 20.361 | 19 | 0.021 |
| WHOQOL psychological | 6 | 0.229 | 0.490 | -0.578 | 0.933 | 0.858 | 11.793 | 18 | 0.733 | 0.929 | 0.770 | 0.147 | 19.485 | 14 | 0.025 |
| WHOQOL total | 24 | -0.150 | 0.739 | -0.718 | 0.990 | 0.946 | 9.547 | 18 | 0.909 | 1.024 | 0.908 | 0.549 | 55.045 | 57 | 0.047 |
|  | ideal values | 0 | <1.4 | 0 | <1.4 | >0.05 Bonferroni corrected |  |  | >0.75 | >0.95 | >0.75 | >0.05 Bonferroni corrected |  |  | <0.05 (lower CI) |

Table S1. Rasch fit statistics. PSI=person separation index, A=expected common variance.

**1.2 Multiple Regression**

The TONiC dataset was used to test the relationship between pain, depression and anxiety on Quality of Life (QoL). In the main text, we employ a bivariate regression model to provide a better understanding of how pain, depression and anxiety affect the different domains of QoL. Previous literature has used overall measures of QoL in multiple regression analyses, thus, we have provided a similar analysis here for comparison.

We analysed the effects of pain, depression and anxiety on overall QoL using the person estimates from the combined domains of the WHOQOL-BREF. Pain, depression, and anxiety all had significant effects on QoL in the univariate analysis (Figure S1). However, anxiety was removed during the process of model selection – it no longer had a significant effect on QoL once pain and depression had been accounted for (Table S2).


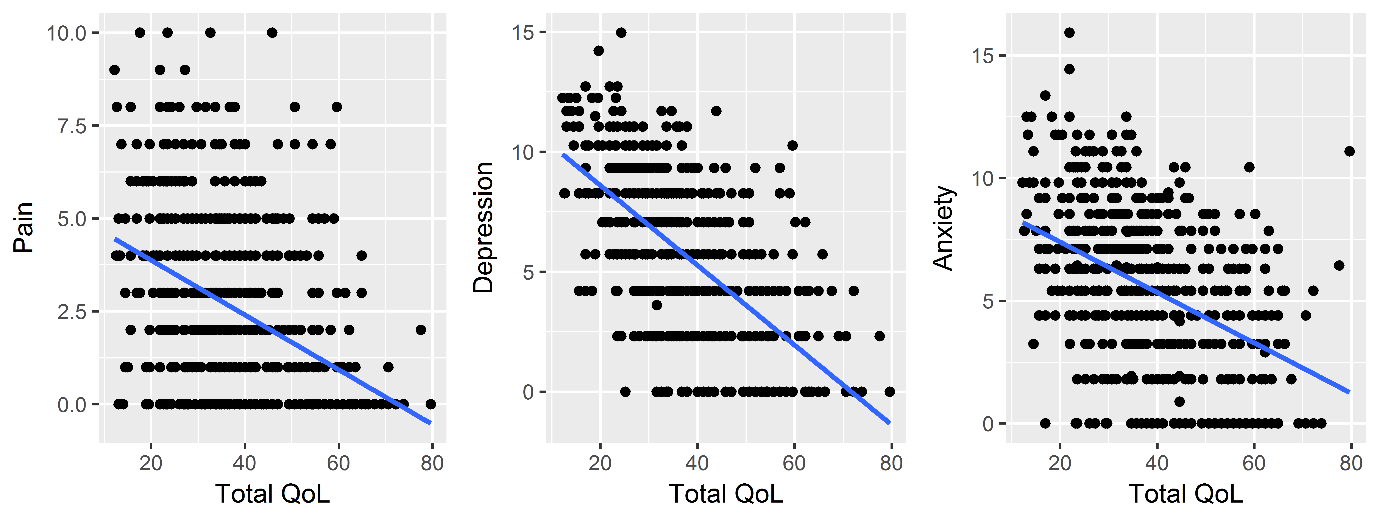


Figure S1: Scatterplots of total QoL against pain, depression and anxiety, the fitted regression line for each univariate analysis is overlaid.

|  | Estimate | Standard Error | t-value | pr(>\|t\|) |
| --- | --- | --- | --- | --- |
| Intercept | 38.567 | 0.4036 | 95.546 | < 2e-16 |
| Pain | -3.5305 | 0.4092 | -8.627 | < 2e-16 |
| Depression | -7.5671 | 0.4052 | -18.676 | < 2e-16 |
| Pain * Depression | 1.2557 | 0.3872 | 3.243 | 0.001 |

Table S2: Standardised parameter estimates for the multiple regression model for total QoL, adjusted R-squared: 0.475, n=597.

**1.3 Further details for the regression model**

The bivariate model accommodates two response variables. In this study, we have two measures for quality of life, psychological and physical quality of life - the responses. Our independent variables are pain, anxiety, and depression. The multivariate model is formulated as (in matrix notation):

$$\boldsymbol{Y}=\boldsymbol{X} \times\boldsymbol{B}+\boldsymbol{E}$$

Where:

- $\boldsymbol{Y=}\left[ \boldsymbol{y}_{\boldsymbol{1}}\boldsymbol{, \ldots,}\boldsymbol{y}_{\boldsymbol{m}} \right]$ is a $n \times m$ response matrix, note: $\boldsymbol{y}_{\boldsymbol{k}}\boldsymbol{=}\left( y_{1k}, \ldots,y_{nk} \right)^{'}$;
- $\boldsymbol{X=}\left[ \boldsymbol{1}_{\boldsymbol{n}}\boldsymbol{,}\boldsymbol{X}_{\boldsymbol{1}}\boldsymbol{,\ldots,}\boldsymbol{X}_{\boldsymbol{p}} \right]$ is a $n \times(p+1)$ design matrix of independent variables;
- $\boldsymbol{B=[}\boldsymbol{b}_{\boldsymbol{1}}\boldsymbol{,\ldots,}\boldsymbol{b}_{\boldsymbol{m}}\boldsymbol{]}$ is a $\left( p+1 \right)\times m$ matrix of regression coefficients and;
- $\boldsymbol{E=[}\boldsymbol{e}_{\boldsymbol{1}}\boldsymbol{,\ldots,}\boldsymbol{e}_{\boldsymbol{m}}\boldsymbol{]}$ is a $n \times m$ matrix of errors, each of which we assume are independent and multivariate normally distributed.

The bivariate model is produced when$m=2$.

The maximum likelihood estimator for B is:

$$\hat{\boldsymbol{b}_{\boldsymbol{k}}}=\left( \boldsymbol{X}^{'}\boldsymbol{X} \right)^{-\mathbf{1}}\boldsymbol{X}^{'}\boldsymbol{y}_{\boldsymbol{k}}$$

Bivariate models are able, through the procedures of statistical inference, to take account of correlations amongst the responses. Thus, they can be more powerful than separate multiple linear regressions for each response variable. The model was fit using R statistical software with relevant packages and results reported using coefficient plots.^11,12^

**Multivariate model assumptions.**

1. The relationship between $\boldsymbol{y}_{\boldsymbol{k}}$ and $\boldsymbol{x}_{\boldsymbol{j}}$ is linear
2. $y_{ik}$and $x_{ij}$ are observed random variables
3. $\left( e_{i1}, \ldots,e_{im} \right)\sim N\left( \boldsymbol{0}_{\boldsymbol{m}}\boldsymbol{,}\boldsymbol{\Sigma} \right)$ is an unobserved random vector.
4. The regression coeffiencents are unknown constants
5. There is homogeneity of variance for each response.

**References**

1 Rasch G. Studies in mathematical psychology: I. Probabilistic models for some intelligence and attainment tests. 1960.

2 Fischer GH, Molenaar IW. Rasch models: Foundations, recent developments, and applications. Springer Science & Business Media, 2012.

3 Masters GN. A rasch model for partial credit scoring. *Psychometrika* 1982; **47**: 149–74.

4 Andrich D, Sheridan B, Luo G. RUMM2030: An MS Windows computer program for the analysis of data according to Rasch unidimensional models for measurement. *Perth Aust RUMM Lab* 2013.

5 Hagquist C, Andrich D. Recent advances in analysis of differential item functioning in health research using the Rasch model. *Health Qual Life Outcomes* 2017; **15**: 181.

6 Wainer H, Kiely GL. Item Clusters and Computerized Adaptive Testing: A Case for Testlets. *J Educ Meas* 1987. https://onlinelibrary-wiley-com.ezproxy.lancs.ac.uk/doi/abs/10.1111/j.1745-3984.1987.tb00274.x (accessed May 2, 2018).

7 Rodriguez A, Reise SP, Haviland MG. Evaluating bifactor models: Calculating and interpreting statistical indices. *Psychol Methods* 2016; **21**: 137.

8 Andrich D. Components of Variance of Scales With a Bifactor Subscale Structure From Two Calculations of α. *Educ Meas Issues Pract* 2016; **35**: 25–30.

9 Lundgren Nilsson Å, Tennant A. Past and present issues in Rasch analysis: the functional independence measure (FIM^TM^) revisited. *J Rehabil Med* 2011; **43**: 884–91.

10 Pomeroy IM, Tennant A, Young CA. Rasch analysis of the WHOQOL-BREF in post polio syndrome. *J Rehabil Med* 2013; **45**: 873–80.

11 Friendly M, Sigal M. Recent advances in visualizing multivariate linear models. *Rev Colomb Estad* 2014; **37**: 261–283.

12 Friendly M, Monette G, Fox J. Elliptical Insights: Understanding Statistical Methods through Elliptical Geometry. *Stat Sci* 2013; **28**: 1–39.
